# Bioethanol from poplar: a commercially viable alternative to fossil fuel in the European Union

**DOI:** 10.1186/1754-6834-7-113

**Published:** 2014-07-29

**Authors:** Jade Littlewood, Miao Guo, Wout Boerjan, Richard J Murphy

**Affiliations:** Department of Life Sciences, Imperial College London, London, SW7 2AZ UK; Department of Plant Systems Biology, VIB, Technologiepark 927, 9052 Gent, Belgium; Department of Plant Biotechnology and Bioinformatics, Ghent University, Technologiepark 927, 9052 Gent, Belgium; Centre for Environmental Strategy, Faculty of Engineering & Physical Sciences, University of Surrey, Guildford, Surrey, GU2 7XH UK

**Keywords:** Cellulosic biofuels, Short-rotation coppice poplar, European Union, Techno-economic, Bioethanol

## Abstract

**Background:**

The European Union has made it a strategic objective to develop its biofuels market in order to minimize greenhouse gas (GHG) emissions, to help mitigate climate change and to address energy insecurity within the transport sector. Despite targets set at national and supranational levels, lignocellulosic bioethanol production has yet to be widely commercialized in the European Union. Here, we use techno-economic modeling to compare the price of bioethanol produced from short rotation coppice (SRC) poplar feedstocks under two leading processing technologies in five European countries.

**Results:**

Our evaluation shows that the type of processing technology and varying national costs between countries results in a wide range of bioethanol production prices (€0.275 to 0.727/l). The lowest production prices for bioethanol were found in countries that had cheap feedstock costs and high prices for renewable electricity. Taxes and other costs had a significant influence on fuel prices at the petrol station, and therefore the presence and amount of government support for bioethanol was a major factor determining the competitiveness of bioethanol with conventional fuel. In a forward-looking scenario, genetically engineering poplar with a reduced lignin content showed potential to enhance the competitiveness of bioethanol with conventional fuel by reducing overall costs by approximately 41% in four out of the five countries modeled. However, the possible wider phenotypic traits of advanced poplars needs to be fully investigated to ensure that these do not unintentionally negate the cost savings indicated.

**Conclusions:**

Through these evaluations, we highlight the key bottlenecks within the bioethanol supply chain from the standpoint of various stakeholders. For producers, technologies that are best suited to the specific feedstock composition and national policies should be optimized. For policymakers, support schemes that benefit emerging bioethanol producers and allow renewable fuel to be economically competitive with petrol should be established. Finally, for researchers, better control over plant genetic engineering and advanced breeding and its consequential economic impact would bring valuable contributions towards developing an economically sustainable bioethanol market within the European Union.

## Background

In the European Union (EU), transportation is responsible for around a quarter of greenhouse gas (GHG) emissions, with road transport accounting for two-thirds of transport-related emissions [1]. The transport sector is perceived to lie at the intersection of energy security and climate change policymaking, therefore the development of a biofuels market is currently recognized by EU governments as an approach for mitigating climate change [2]. The Renewable Energy Directive (2009/28/EC) states that by 2020, 10% of transport fuel should come from renewable sources and, furthermore, lignocellulosic biofuels derived from non-food sources will receive double credits to provide an additional incentive for producers [3]. Although the share of biofuels in road transport quadrupled between 2004 and 2007, statistics demonstrate that there is a significant gap between bioethanol production and capacity, and overall consumption has repeatedly fallen short of EU targets [4]. It is therefore evident that EU bioethanol expansion is constrained by various technological, economic or political bottlenecks.

Concerns regarding GHG savings, land-use change and rising food prices associated with first-generation biofuels derived from food crops, have resulted in research and policy support favoring production of lignocellulosic (second-generation) biofuels. However, the ability of biofuels to positively contribute to climate change mitigation continues to be a hotly contended issue [5]. Although environmental assessments of the potential GHG savings achieved from biofuels vary (particularly in relation to the controversial issue of land-use change) the majority indicate that second-generation biofuel production from sustainably sourced lignocellulosic resources would indeed lead to lower overall GHG emissions when compared with first-generation biofuels and fossil alternatives [5]. Poplars (*Populus spp.*) have been the subject of significant interest due to their: 1) potential for management under short- (including very short) rotation coppice (SRC) harvest cycles, 2) wide genetic diversity and available genome sequence, 3) strong heterosis in interspecific hybrids, 4) low nutrient demand and 5) high biomass yield on different types of land [6, 7]. Poplars are important forestry and SRC species in Europe with about 950,000 ha of poplar plantation and 130,000 ha of natural forests with indigenous poplar [8]. This interest has been supported by a number of European Commission and nationally-funded projects on poplars for biofuel, with the present work being undertaken within the Commission of the European Committee’s (CEC) Seventh Framework Programme (FP7) Project EnergyPoplar (FP7-211917).

Due to the natural recalcitrance of lignocellulose, a pretreatment stage is considered necessary to achieve the high sugar release believed to be essential for economic success [9]. Pretreatment technologies applied to poplar include dilute acid (DA), liquid hot water (LHW), ammonia fibre expansion, sulphur dioxide steam explosion and lime, some of which have achieved sugar yields of up to 90% of the original cellulose content [9–13]. Although pretreatment adds to costs within the biochemical conversion process, the low sugar yields achieved in the absence of pretreatment result in greatly reduced ethanol yields, thereby effectively negating the savings achieved by the removal of this step [9]. It has been stated that ‘the only operation more expensive than pretreatment is no pretreatment’; and that an economically feasible process is only attained through minimizing pretreatment costs whilst maximizing sugar yields [9]. The presence of lignin in plant cell walls is understood to not only impede enzyme accessibility to cellulose, but to also reduce the relative amount of cellulose present on a mass balance basis [14–16]. However, this does not mean that reducing lignin results in higher cellulose biosynthesis [16–19]. Modern molecular biotechnology offers alternative approaches to conventional plant breeding techniques for enhancing the chemical composition of biofuel feedstocks, including the altered expression of genes involved in the biosynthesis of the *p*-hydroxyphenyl, guaiacyl and syringyl building blocks of lignin [14, 19, 20], or the engineering of entirely new lignin structures using synthetic biology [21, 22]. Recent studies have successfully demonstrated that by reducing the lignin content in poplar and other woody species, improved accessibility is achieved during enzymatic saccharification [14, 19, 20, 23–26]. However, the impact that these modifications may have from an economic perspective is currently unknown.

Though there are several techno-economic assessments carried out on poplar-to-bioethanol processes, these are limited to variations of feedstock and process design [27–29]. Here, we evaluate the potential for bioethanol production from SRC poplar in five EU countries from technology, economic and policy standpoints, thereby providing a coherent framework to facilitate dialogue between scientists, economists and policymakers on addressing the major bottlenecks within the supply chain, which currently hamper the commercial viability of bioethanol from poplar in the EU.

## Results and discussion

### Effect of technology and national prices on bioethanol production cost

**Figure 1:**
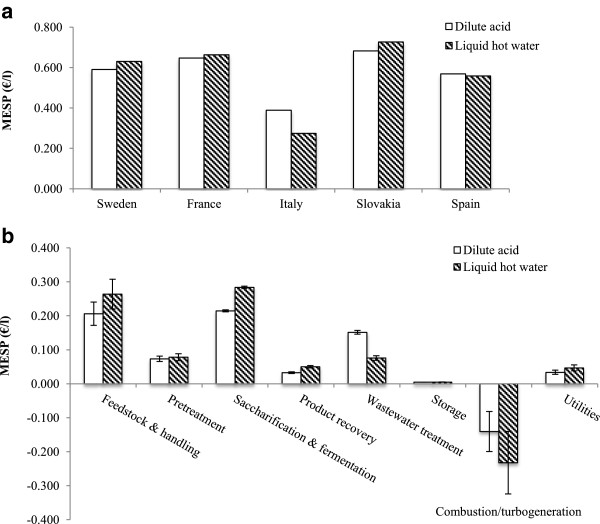
**Net production MESPs and cost breakdown for bioethanol from SRC poplar by LHW (pattern) and DA (white) pretreatment processes in five European countries. (a)** MESPs reflect final cost in each country after inclusion of credits from electricity generation. **(b)** MESP cost breakdowns are averaged from five countries. Error bars represent standard error for the five countries. Negative bars in combustion/turbogeneration reflect negative cost (profit) gained from electricity (after plant demand is satisfied). (MESP, Minimum ethanol selling price; SRC, short-rotation coppice; LHW, Liquid hot water; DA, dilute acid).

While the minimum ethanol selling prices (MESPs) of net bioethanol production for three out of the five countries (excluding Italy and Spain) show that the DA pretreatment process is marginally more economically favorable than the LHW process, these differences should not be over-emphasized due to uncertainties inherent in the baseline assumptions (Figure 1a). The total capital investment of the DA pretreatment process was lower than the LHW pretreatment process at €337 million compared with €345 million for a plant operating at 2,000 dry metric tonnes per day. The MESP cost breakdown presented as an average of the five EU countries (Figure 1b) reveals that the highest cost stages are feedstock and handling, and saccharification and fermentation, with raw materials serving as the single greatest contributor. In feedstock and handling, poplar purchase accounts for 92% of the total cost, whereas enzyme purchase is responsible for 84% of the saccharification and fermentation cost. This clearly demonstrates the importance for the economic viability of poplar bioethanol of accessing cheaper biomass costs as well as reducing enzyme loading in saccharification. The lower sugar conversion efficiencies in LHW pretreatment result in higher enzyme loadings required in saccharification and fermentation to drive sugar release, as well as an increased amount of undigested biomass being sent to the combustor, ultimately inflating the unit MESP for this process.

Italy and Spain are the only countries to have a higher MESP for pretreatment with DA than LHW; Italy also has the lowest overall MESPs for both pretreatments at €0.275/l for LHW and €0.389/l for DA, respectively (Figure 1a). Despite having relatively low costs for ash disposal, labor, electricity credit and income tax, Slovakia still has the highest MESP for both processes at €0.727/l (LHW) and €0.683/l (DA), due to its expensive delivered feedstock cost which, at €80/dry tonne, which is the highest amongst these countries. The lowest feedstock cost of €33/dry tonne of delivered biomass is found in Sweden – considering this, the MESPs for both DA and LHW processes are unusually high; this is attributed to the relatively low credit obtained in Sweden for the co-produced electricity (€0.033/kWh).

Major support systems for renewable electricity generation in the EU include feed-in tariffs, quota obligations, feed-in premiums and tradable electricity certificates. Of these, feed-in tariffs are considered to be the most effective mechanism by guaranteeing a fixed price per kWh of electricity sold back to the grid [30]. Of the five countries, Sweden is the only one to use a combination of electricity certificates and quota obligation to reach its goal of 17.9% share of renewable electricity by 2011 [31]. In Sweden, renewable electricity producers receive certificates of a fixed price, which can be sold to receive income for their production [31]. Compared to Italy’s high credit of €0.25/kWh, or even to Spain, Slovakia and France, which all employ feed-in tariffs ranging from €0.12 to €0.18/kWh, electricity producers in Sweden obtain little for their electricity. Under the LHW process which generates 35 MW more electricity than the DA process, the difference in the value of the electricity credit between Sweden and Italy for the plant of the scale modeled (2,000 oven-dry tonnes feedstock processed per day) is equivalent to approximately €64 million per annum, thereby demonstrating the importance the electricity credit can have on the overall cost structure of bioethanol production. In Italy, the combination of a very high feed-in tariff with a relatively low poplar price results in the most economically favorable situation for producing bioethanol from poplar.

Other costs and regulations including ash disposal, labor and income tax rates play relatively minor roles in their contributions towards the MESPs. Although feedstock and enzyme prices as well as electricity credits are the principal drivers of bioethanol production, additional factors such as excise duties and distribution costs are significant and can double the final ethanol selling price at the pump (petrol filling station) compared with its initial production cost.

### Influence of policy support on the bioethanol pump price

The DA and LHW pretreatment processes are compared against national Euro Super-95 petroleum prices in 2011 to assess the competitiveness of bioethanol derived from SRC poplar in the EU countries [32]. The fuel price at the pump consists of its production cost, a fuel excise tax and a value-added tax (VAT) on the sum of the total production cost plus the excise tax combined. It has been recognized that governmental support schemes for bioethanol may be the single largest factor dictating its competitiveness with petrol at the pump [2, 4]. Production quotas, mandatory blending targets and fiscal incentives in the form of tax reliefs are the main types of policy support applied to biofuels within the EU [2, 4]. Of the 27 Member States, 20 States provide either full or partial tax exemption for each liter of biofuel supplied on the market (9 provide full tax relief on bioethanol) [4].

**Figure 2:**
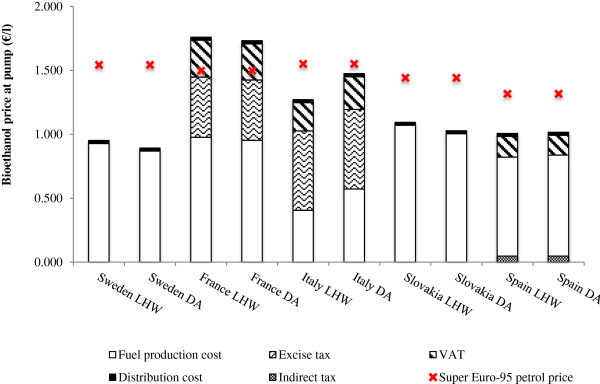
**Comparison of SRC poplar bioethanol pump price with petrol.** All prices are reported as €/l in 2011 [32]. National petrol prices are marked by the red cross. (SRC, short-rotation coppice; LHW, liquid hot water; DA, dilute acid; VAT, value-added tax).

Sweden and Slovakia provide full exemption from taxes, allowing bioethanol to be competitive with petrol under both DA and LHW processes (Figure 2). Sweden would also have the lowest bioethanol pump price for both LHW and DA processes at €0.952/l and €0.893/l, respectively. Although VAT and indirect taxes are imposed on bioethanol in Spain, its inexpensive poplar price combined with the lowest VAT rate (18% in 2011) means that SRC poplar bioethanol would still be competitive with petrol and has the second lowest price at the pump of the five countries. Despite having the lowest production costs (Figure 1a), lack of government subsidy in Italy means its pump price is more than doubled over its initial production cost, surpassing bioethanol prices in Sweden, Slovakia or Spain [33]. Regardless of this, bioethanol in Italy under both pretreatment processes is still competitive with petrol due to its low feedstock price and high electricity credits. Bioethanol production in France would be subject to the full VAT rate, but has a partial tax exemption of €0.14/l which is applied only to a certain quota of ethanol (in 2010 this was 867,000 tonnes). As a result, France has the highest SRC poplar bioethanol pump prices of €1.760/l (LHW) and €1.732/l (DA), both which are higher than petrol (€1.498/l). As a result of these differing support schemes, there is wide variability in the theoretical pump prices of SRC poplar bioethanol amongst these EU countries.

The competitive advantage that full tax exemption offers bioethanol producers is obvious in these results: without such exemptions poplar bioethanol prices would not be competitive with petrol in Sweden or Slovakia. Although SRC poplar bioethanol production in France is subject to partial tax relief and a production quota, each year this quota is increased with a successive decrease in tax relief to minimize the amount of fiscal losses for the government and the burden on taxpayers [4]. In 2008, the estimated cost of excise tax exemption in France was €168 million, which is the second highest in the EU after Sweden (€224 million) [4]. In the short-term, government support is likely to remain necessary to enable new bioethanol production from ‘second-generation’ sources, such as locally-produced SRC poplars, to emerge. However, as the industry matures, it is probable that support such as tax reliefs will be scaled back, emphasizing the need to seek out alternative approaches to sustain and promote the long-term growth of the second-generation bioethanol market.

### Prospects for improved and advanced SRC poplar

Our prospective case is modeled on the work of the CEC FP7 EnergyPoplar project and literature [34]. It uses a poplar SRC feedstock with projected lignin-modifications (induced by genetic engineering and/or advanced breeding science), which display predicted and laboratory-demonstrated improvements for downstream processing. Under this prospective scenario - here termed the ‘EnergyPoplar’ scenario - the MESP is reduced in four countries by approximately 41% from the base-case MESPs (Table 1). In Italy, however, the high electricity tariff (which favors electricity generation and hence higher lignin contents), results in a somewhat lower 31% improvement in the MESP from the DA process, and only a 6% improvement from the more economically favorable LHW process.

**Figure 3:**
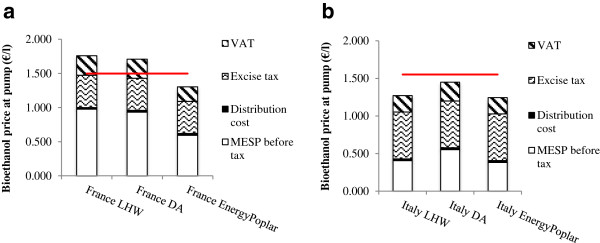
**Bioethanol pump price for prospective and base-case processes against petrol in (a) France and (b) Italy.** Euro Super-95 prices (in 2011) at the pump are indicated by the red mark [32]. (LHW, liquid hot water; DA, dilute acid; MESP, minimum ethanol selling price; VAT, value-added tax).

In countries where bioethanol production is fully exempt from tax (Sweden and Slovakia) bioethanol pump prices would already be competitive with petrol, so the EnergyPoplar scenario enhances this competitiveness. For countries where bioethanol is either not competitive, or has very little advantage over petrol (France and Italy), even with low or zero levels of government support, the improvements to the SRC poplar feedstock would allow bioethanol from EnergyPoplar SRC poplars to become competitive with petrol at the pump (Figure 3).

**Table 1:** **Comparison of MESPs for the prospective EnergyPoplar case per country with base-case processes using LHW and DA pretreatments. (MESP, minimum ethanol selling price; LHW, liquid hot water; DA, dilute acid)**

	MESP (€/l)		
	LHW	DA	EnergyPoplar scenario (prospective)
Sweden	0.631	0.591	0.318
France	0.664	0.648	0.404
Italy	0.275	0.389	0.259
Slovakia	0.727	0.683	0.418
Spain	0.559	0.569	0.346

The EnergyPoplar scenario cost savings arise from two main areas. It was demonstrated experimentally by Mansfield *et al*. that by suppressing a specific gene involved in cell wall lignification (*p*-coumarate 3’-hydroxylase (C3’H)), greater enzyme accessibility in poplar trees could be achieved without any form of pretreatment [23]. Therefore, the removal of the pretreatment stage in our process design led to substantial savings in both capital and raw material expenditure. Secondly, it was also shown to be possible to reach glucose yields of 80% in enzymatic saccharification with low enzyme loadings of 10 filter paper units (FPU)/g glucan, thereby significantly reducing enzyme purchase costs, particularly in comparison to the high-enzyme loading LHW process scenario [23]. While not modeled in the current work, a more recent study by Van Acker *et al*. also demonstrated an alternative method of modifying lignin content in poplars that could enhance ethanol yields after simultaneous saccharification and fermentation [26]. By reducing expression of cinnamoyl-CoA reductase (CCR) (an enzyme involved in the monolignol-specific branch of lignin biosynthesis) ethanol yields in the most severely affected trees yielded up to 161% more ethanol than the wild-type trees [26].

One concern that should not be overlooked however is the relationship between lignin content and growth phenotype. This phenomenon was observed in both studies [20, 23, 26], such that trees with lower levels of lignin were substantially smaller than their wild-type counterparts. It is well-known that lignin plays an essential part in plant cell wall structure by providing support, water transport and defense against microbial and enzymatic attack, therefore it is crucial to ensure that a reduction in lignin content is not associated with a decrease in plant survival or health [20, 34–36]. Mansfield *et al*. [23] measured the tree volume of eight-month-old glasshouse grown transgenic poplar trees and found that the C3’H-14 line with the lowest lignin content used in their study had a volume reduction of 73% compared with the wild-type. Van Acker *et al*. also revealed a similar finding with CCR-down-regulated trees, in that poplar lines with reduced lignin contents had reductions in biomass yield, diameter and height [26]. When this biomass yield penalty was accounted for, ethanol yields were significantly reduced in many of the transgenic lines. In the situation where trees with enhanced biomass properties for biofuel production are found to produce less biomass per plant, the potential economic ramifications need to be seriously considered. For example, in order for an equal amount of biomass to be produced on a volume basis, trees may have to either be planted at higher densities, or occupy greater areas of land. This may impact feedstock prices, possibly negating the savings achieved from capital and enzyme cost reductions.

Despite these downsides, modern biotechnology is focused on avoiding problems associated with reducing lignin content, as well as identifying alternative routes to produce plants with improved phenotypic traits for biofuels. Firstly, the aforementioned diminished biomass yields can be related to the effect that low lignin content has on xylem vessels, whether this be due to the collapse of vessels and/or increased embolism from entry of air bubbles in water-conducting cells, or formation of tyloses which block vessels and reduce water-transport efficiency [37]. Current research aims to resolve this by using promoters to drive transgene expression in fibres only, as a strategy to concentrate lignin reduction in fibres and not in vessels [15, 35]. In addition to using tissue specific promoters to reduce lignin in fibres only, engineering signaling pathways responding to cell wall defects may help reduce the yield penalty typically associated with lignin engineering. Secondly, rather than down-regulating lignin biosynthesis, but instead by altering the composition of existing lignin, (for example, *p-*hydroxyphenyl/guaiacyl/syringyl unit ratios, or cinnamaldehyde/cinnamyl alcohol ratios in lignin) improved susceptibility of the polymer to degradation during pretreatment and/or saccharification has also been achieved [14]. Finally, whilst the production of transgenic trees is a rapidly evolving area within this field, the potential for such improvements that is provided by advanced poplar breeding programs should also not be overlooked. The vast genetic variability in composition and saccharification potential of poplars [38] means that the screening and sexual crossing of genotypes with desirable characteristics to yield an F1 generation with elite genotypes for baseline material and for further genetic modification, is a very promising route for the future of the biofuel industry.

## Conclusions

In order for bioethanol to contribute towards the EU climate-change-driven target of achieving a 10% renewable energy share in the transport sector by 2020, its production must be both commercially viable for the producer, as well as economically competitive for the consumer. The technological, economic and political bottlenecks within the bioethanol supply chain have been addressed in this study, highlighting the main priorities for producers, policymakers and researchers to commercialize a SRC poplar-derived bioethanol process within the EU.

Irrespective of the processing technology, feedstock and enzyme prices have consistently emerged as key determinants influencing the cost of bioethanol production. Producers should therefore focus on utilizing a pretreatment technology which is cost-effective yet maximizes sugar yields, so as to reduce the downstream enzyme contribution towards the total cost of production. Additionally, producers must consider accessing inexpensive feedstock costs and seek benefit by establishing second-generation bioethanol production projects in countries having support mechanisms and timescales that sustain lower cost production of feedstock.

For policymakers, implementation of cost-effective policies (such as waste disposal, income tax and electricity credits) and schemes to support emerging producers, can be driving factors determining the economic competitiveness of SRC poplar bioethanol with petrol. Policies regulating electricity generation credit (whether as tariffs or certificates) vary between the EU countries, and (as seen in Italy) have potential to provide bioethanol cost reductions, especially in selecting optimal processing technologies. Fiscal incentives such as exemptions from VAT and excise duties are the major support systems used by governments, which are effective in offering a major competitive advantage to bioethanol (as seen in Sweden and Slovakia). However, for countries where costs are low and co-product credits are high, bioethanol production can be competitive at reduced levels of government intervention, offering a potentially more economically sustainable situation for the long-term.

Finally, for researchers, the understanding of feedstock composition and cell wall accessibility is shown here to be essential to making bioethanol production economically viable. Recent studies in poplar development and biomass modification indicate the realistic potential to design poplar lines releasing high levels of sugar without requirement for pretreatment and with low demand for processing enzyme loadings. Our results demonstrate the valuable commercial impact that genetic engineering and/or improvement of poplars (for example, to produce a significantly reduced lignin content) can have on reducing the costs of bioethanol production. However, it is recognized that wider phenotypic aspects of ‘advanced’ poplars (such as biomass yield, stability, resistance to disease and pests) will require confirmation in order to capitalize on this new trait of improved biofuel conversion.

This work shows that contributions from science and technology, economics and policy all influence the potential to develop a viable SRC poplar-based bioethanol market within the EU. Sustaining the development of advanced poplar feedstocks and processing technology for economically viable biofuel production within the EU will provide climate mitigation benefits within the technological, economic and environmental constraints that apply.

## Methods

### Process design parameters

The composition of poplar and the key parameters for its pretreatment and subsequent enzymatic saccharification were derived from empirical data from published experimental work and publically available databases. These data were used as input for a process design model using AspenPlus™ software (Aspen Technology, Inc., Massachusetts, United States) to generate mass and energy balances for the economic analysis. Sweden, Italy, Slovakia, Spain and France were selected as the five European countries representing different regions of Europe.

### Poplar composition

The baseline poplar composition was derived from the National Renewable Energy Laboratory (NREL) database for Hybrid Poplar Caudina (*Populus deltoides* x *Populus nigra* var. *caudina*) [39]. Compositions of the baseline poplar biomass and the EnergyPoplar prospective scenario with reduced lignin content are provided in Table 2. Detailed methodology for generating the transgenic poplar line is described in [34] (See footnote of Table 2).

**Table 2:** **Compositions of hybrid poplar and genetically lignin-modified poplar biomass**

	Glucan	Xylan	Galactan	Arabinan	Mannan	Lignin	Ash	Extractives	Reference
Hybrid Poplar Caudina	45.3%	15.5%	1.0%	1.0%	2.1%	28.2%	2.0%	5.0%	[39]
Genetically lignin-modified poplar (C3’H-14)^a^	55.1%	22.8%	1.0%	0.5%	1.8%	11.3%	2.2%	5.4%	[34]

^a^This lignin modified poplar is down-regulated for *p*-coumarate 3’-hydroxylase, an enzyme involved in lignin biosynthesis. The genetically modified variety has 56% less lignin as compared to its non-modified control [34] _._

### Pretreatment and enzymatic saccharification

Pretreatment and subsequent enzymatic saccharification work conducted by the Biomass Refining Consortium for Applied Fundamentals and Innovation (CAFI) team evaluating the effects of DA and LHW (controlled pH) pretreatment technologies on poplar wood [9] was used in this model. The conditions, reactions and yields for these processes are listed in Table 3. For the data not provided in the literature (listed as NA in Table 3), assumptions were made to complete the process design. It was assumed that no glucan or xylan is converted into glucose or xylose oligomers in the DA pretreatment. It was also assumed that the conversion yields of xylan into xylose would reflect the conversion yields for C5 sugars into their monomeric components, and that the conversion of glucan into glucose would represent the yields for C6 sugars into their monomers, during the pretreatment and enzymatic saccharification stages. In the prospective EnergyPoplar scenario (with reduced lignin levels via genetic modification), glucose yields of 80% were assumed to be achieved without pretreatment [23]. For the EnergyPoplar scenario, the pretreatment area was removed and enzymatic saccharification was performed with a reduced enzyme loading. The sugar yields from enzymatic saccharification from polysaccharides were assumed to be the same as the glucose yield (empirical data available in literature) to represent what could potentially be achieved for the future scenarios [23]. Other process conditions such as total solids loading in the EnergyPoplar scenario were adopted from the LHW and DA pretreatment processes, and these assumed that future advances in chemical engineering process design would be able to achieve comparable yields at similar design parameters.

**Table 3:** **Summary of pretreatment and enzymatic saccharification conditions and results**

Process scenario	Liquid hot water (LHW)	Dilute acid (DA)	EnergyPoplar scenario (prospective)
Pretreatment conditions	200°C for 10 minutes	190°C for 1.1 minutes, 2.0% H_2_SO_4_	NA
Pretreatment reactions	Fraction of reactant converted to product		NA
Glucan + H_2_O → Glucose	2%	24%	NA
Glucan → Glucose oligomers + H_2_O	2%	NA	NA
Xylan + H_2_O → Xylose	4%	62%	NA
Xylan → Xylose oligomers + H_2_O	54%	NA	NA
Arabinan + H_2_O → Arabinose	NA^a^	NA	NA
Mannan + H_2_O → Mannose	NA	NA	NA
Galactan + H_2_O → Galactose	NA	NA	NA
Lignin → Soluble lignin	25%	NA	NA
Enzymatic saccharification conditions^b^	(15 FPU cellulase + 40 CBU β-glucosidase)/g glucan in original biomass for 72 hours	(15 FPU cellulase)/g glucan in original biomass for 72 hours	(10 FPU cellulase)/g glucan in original biomass for 72 hours
Enzymatic saccharification yields	55.0% glucose yield, 89.8% xylose yield	82.5% glucose yield, 24.7% xylose yield	80.0% glucose yield, 80.0% xylose yield
References	[9, 13]	[9]	[23]

^a^NA = Data not available.

^b^FPU, Filter paper units; CBU, Cellobiase units.

### AspenPlus™ process simulation

**Figure 4:**
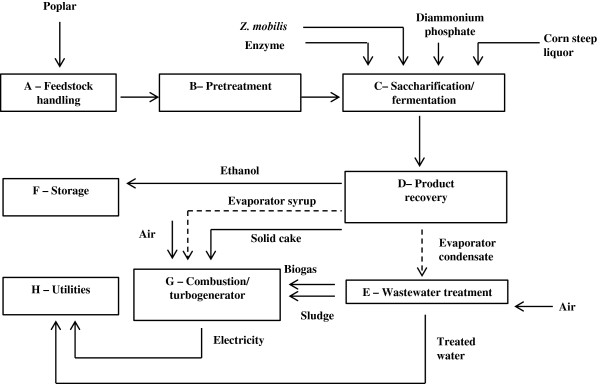
**Schematic diagram of poplar-to-bioethanol process.** Dashed lines from Area D represent alternative routes for the distillation column bottoms in dilute acid and liquid hot water processes.

The techno-economic process design was adapted from the NREL model [40], designed to process 2,000 dry metric tonnes of poplar biomass per day. An overview of the main process areas is shown in the schematic diagram in Figure 4.

Poplar is unloaded and unwrapped at the feedstock handling (Area A in Figure 4) where it is washed and milled to a suitable particle size. It is then conveyed to pretreatment (Area B) where it undergoes LHW and DA pretreatment at a total solids loading of 30% (w/w) [40]. Under DA pretreatment, an additional neutralization step with ammonia is required to raise the hydrolysate pH to 5 before enzymatic saccharification. Pretreated poplar is sent to separate saccharification and fermentation (Area C) to enzymatically hydrolyse polysaccharides into monomeric sugars and then ferment them into ethanol using the fermentative bacterium, *Zymomonas mobilis*. Saccharification is carried out at 50°C for 72 hours. The hydrolysate is cooled to 32°C and a portion is sent to two *Z. mobilis* seed inoculation trains with a residence time of 24 hours each, and the rest is sent to fermentation tanks operating for 36 hours. The *Z. mobilis* strain in this design is a recombinant microorganism chosen for its ability to ferment both hexose and pentose sugars. Nutrient loadings (corn steep liquor (CSL) and diammonium phosphate (DAP)) and fermentation sugar conversion efficiencies (95% of glucose, 85% of xylose and arabinose) are adopted from the NREL process [40]. Of the monomeric sugars, 3% are assumed to be converted into glycerol, succinic acid and xylitol due to contaminations [40]. The fermentation beer is sent to product recovery (Area D) where ethanol is concentrated through distillation and molecular sieve adsorption to 99.6%. Distillation bottoms obtained from the distillation column contain unfermented monomeric sugars, organic acids and solid residuals such as lignin, extractives and ash. These distillation bottoms from the different pretreatments are dealt with in two ways: (1) Under LHW pretreatment, a series of evaporators are used to produce a condensed syrup from the soluble organics, and a lignin-rich solid cake which, when combined, has a moisture content below 45%. This is sent to the combustor/turbogenerator (Area G) for steam and electricity generation. (2) Under DA pretreatment a press filter is used to separate solids from liquids. The solid residue is directed to the combustor and liquids are sent to wastewater treatment (Area E).

The wastewater treatment area includes anaerobic and aerobic digestion which treats and recycles used water within the process to minimize the amount of water discharged to the environment and the purchased fresh water requirement. In anaerobic digestion, 91% of organic matter is converted into biogas and microorganism cell mass. The biogas with a composition of 51% CH_4_/49% CO_2_ (w/w) is assumed to be produced at a yield of 228 g biogas/kg chemical oxygen demand (COD) removed. The treated water is then cleaned in aerobic digestion where 96% of the remaining soluble organic matter is removed. Where ammonia is required for acid neutralization in DA pretreatment, caustic soda is used to remove accumulated ammonium salts as sodium nitrate; this is modeled as brine waste and sent to landfill [40].

The concentrated syrup is combined with the solid cake from the distillation bottoms, biogas and cell mass (sludge) obtained from wastewater treatment and this is fed to the combustor (Area G) for combined heat and power (CHP) generation. High-pressure steam is extracted from the turbine to meet process heat requirements. The generated electricity supplies the process energy demands, and it is assumed that surplus electricity is sold to the National Grid as a co-product credit. Under DA pretreatment, the flue gas released from the combustor requires desulphurization by applying limestone before being emitted to the atmosphere.

The utilities area (Area H) includes the cooling tower, clean-in place and plant air systems. Feedstock, chemicals and products are stored in the storage area (Area F).

### Process economics

From the generated mass and energy balances in AspenPlus™, the Total Capital Investment (TCI) was determined from equipment purchased and installation costs estimated from process specifications. Equipment costs were derived from NREL’s vendor quotations. These reflect a baseline equipment size which was scaled up or down according to the exponential scaling expression (Equation 1) [40], where the *f* scale represents a scaling exponent specific for each piece of equipment, usually ranging between 0.6 and 0.7:
1

All costs were indexed using the Chemical Engineering Plant Cost Index to the reference year of 2011 chosen for this study [40]. Direct and indirect costs were added together to yield the TCI. Direct costs included warehouse, site development and additional piping, comprising 4%, 9% and 4.5% of the inside-battery-limits (ISBL) equipment costs (Areas B to D), respectively. Indirect costs included proratable costs (10% of total direct cost), field expenses (10%), home office and construction (20%), project contingency (10%) and other costs (10%) [40].

The raw material costs contribute to the variable operating costs and are only incurred while the process is in operation; these are listed in Table 4. Fixed operating costs include labor and various overhead items which are incurred whether or not the plant is producing at full capacity. Annual maintenance materials are estimated as 3% of the ISBL capital cost, and local property tax and property insurance are assumed to be 0.7% of the fixed capital investment [40].

**Table 4:** **Summary of raw material costs**

Inputs	Price	Reference
Sulphuric acid	64.6 €/tonne	[41]
Ammonia	342.0 €/tonne	[40]
Lime (Ca(OH)_2_)	152.0 €/tonne	[40]
Corn steep liquor	43.3 €/tonne	[40]
Diammonium phosphate (DAP)	462.1 €/tonne	[42]
Enzyme	379.2 €/tonne	[43]
Sorbitol	858.8 €/tonne	[40]
Caustic	372.4 €/tonne	[44]
Fresh water	0.20 €/tonne	[40]
Boiler feed water chemicals	3808.6 €/tonne	[40]
Cooling tower chemicals	2720.4 €/tonne	[40]

Other cost parameters (Table 5) required in the analysis are country-specific, including feedstock cost, or have been established according to government policy (such as waste disposal charges, electricity credit and income tax). The country-specific cost and price parameters constantly fluctuate with time. The reference year 2011 was adopted in this study to evaluate the potential for poplar-derived bioethanol production in EU countries and to address the major bottlenecks within the potential bioethanol supply chains. The number of employees has been adopted from Humbird *et al*. [40] Baseline salaries are derived from a study based in the United Kingdom, and labor ratios for each country are calculated according to the average salary of each European country relative to the United Kingdom in 2011 [45, 46].

**Table 5:** **Summary of cost and fuel price parameters (2011) in five European countries**

	Sweden	France	Italy	Slovakia	Spain	Reference
Cost parameters						
Delivered feedstock price (€/odt)^a^	33.3	75.6	34.3	83.8	57.0	[47–50]
Landfill tax (€/tonne)	62.5	20.0	86.5	1.7	32.7	[51]
Electricity credit^b^ (€/kWh)	0.033^c^	0.13^d^	0.25^d^	0.12^d^	0.13^d^	[52]
Income tax	26.3%	34.4%	27.5%	19.0%	30.0%	[53]
Labor ratio	1.95	1.70	1.33	0.42	1.02	[45, 46]
Fuel price parameters 2011 (€/l)						
Euro Super-95 pump price in 2011	1.543	1.498	1.551	1.443	1.317	[32]
Fuel excise tax	0.612	0.611	0.622	0.551	0.425	[32]
Value-added tax (VAT)	25%	19.6%	21%	20%	18%	[32]
Bioethanol support policy	Fully exempt	Partially exempt (€0.14/l)	Not exempt	Fully exempt	Exempt from hydrocarbon tax but not VAT, and subject to an indirect tax on the retail sales of certain hydrocarbons (additional national €0.024/l and regional rate of €0.024/l)	[2, 32, 33, 54]

^a^Includes a transportation distance of approximately 50 km [55] (Odt, oven-dry tonne).

^b^Credit refers to the amount that renewable electricity generators can receive from selling their excess electricity to the grid or other suppliers and/or distributors.

^c^Price of electricity certificate given to renewable electricity producers per MWh of electricity generated.

^d^Electricity from renewable sources is promoted through a price regulation system based on a fixed feed-in tariff.

### Discounted cash flow analysis

Once TCI and operating costs were determined, a discounted cash flow method was used to estimate the minimum ethanol selling price (MESP). This refers to the bioethanol price at which the net present value of the project is zero at a set discount rate of 10%. This model is based on ‘n^th^ plant’ assumption which assumes several plants using the same technology are currently operating, eliminating additional costs associated with pioneer plants [40]. The parameters used in the discounted cash flow calculation are listed in Table 6. To investigate the commercial viability of bioethanol in different EU countries and to emphasize the variability in bioethanol selling prices as a result of different processing technologies, consistent assumptions for distributing the cost over their life cycles in the supply chains modeled were used (Table 6). Thus, the same accounting methods (based on the MACRS approach used in the NREL study [40]) were adopted for modeling these EU poplar-derived bioethanol supply chains in the current study. Although the accounting methods between EU countries and MACRS may vary, this is beyond the scope of current research and could be further investigated in future research.

**Table 6:** **Discounted cash flow assumptions**

Parameters	Value
Plant life	30 years
Discount rate	10%
Financing	40% equity
Loan terms	10-year loan at 8% APR
General plant depreciation	200% declining balance^a^
General plant recovery period	7 years
Steam plant depreciation	150% declining balance
Steam plant recovery period	20 years
Construction period	3 years
0-12 months	8% of project cost
12-24 months	60% of project cost
24-36 months	32% of project cost
Working capital	5% of fixed capital investment
Start-up time	3 months
Revenues during start-up	50%
Variable costs incurred during start-up	75%
Fixed costs incurred during start-up	100%

^a^Depreciation method is the IRS Modified Accelerated Cost Recovery System (MACRS). APR, annual percentage rate.

### Supply-chain model

A supply-chain model was established to determine the bioethanol price at pump for comparison with petrol using a reference year of 2011. This price includes the bioethanol production cost, fuel duty, VAT, a feedstock transportation cost and a fuel distribution cost. The energy content of bioethanol (21.2 MJ/l) is less than petrol (31.2 MJ/l); 1 liter of bioethanol is therefore equivalent to 0.68 liters of petrol. All fuel prices have thus been adjusted to take into account these differences in energy content. It was assumed that poplar is transported by lorry from a distance within 50 km of the bioethanol plant. An average transportation and handling charge of €0.070/km/tonne of poplar was adopted from Neuvonen, 2010 [55].

## Abbreviations

CHP: Combined heat and power
COD: Chemical oxygen demand
CSL: Corn steep liquor
DA: Dilute acid
DAP: Diammonium phosphate
GHG: Greenhouse gas
ISBL: Inside-battery-limits
LHW: Liquid hot water
MESP: Minimum ethanol selling price
NREL: National Renewable Energy Laboratory
SRC: Short-rotation coppice
TCI: Total capital investment
VAT: Value-added tax.
